# *NBN* gain is predictive for adverse outcome following image-guided radiotherapy for localized prostate cancer

**DOI:** 10.18632/oncotarget.2404

**Published:** 2014-08-27

**Authors:** Alejandro Berlin, Emilie Lalonde, Jenna Sykes, Gaetano Zafarana, Kenneth C. Chu, Varune R. Ramnarine, Adrian Ishkanian, Dorota H.S. Sendorek, Ivan Pasic, Wan L. Lam, Igor Jurisica, Theo van der Kwast, Michael Milosevic, Paul C. Boutros, Robert G. Bristow

**Affiliations:** ^1^ Departments of Radiation Oncology, Medical Biophysics, Medical Oncology, Laboratory Medicine and Pathology, Pharmacology & Toxicology and Biostatistics, Computer Science, University of Toronto, Toronto, ON, Canada; ^2^ Radiation Medicine Program, Princess Margaret Cancer Centre, University Health Network, Toronto, ON, Canada; ^3^ Informatics and Bio-Computing, Ontario Institute for Cancer Research, Toronto, ON, Canada; ^4^ Vancouver Prostate Centre & Department of Urologic Sciences, University of British Columbia, Vancouver, BC, Canada; ^5^ Department of Integrative Oncology, British Columbia Cancer Research Centre, Vancouver, BC, Canada; ^6^ The Techna Institute, University Health Network, Toronto, ON, Canada

**Keywords:** prostate cancer, NBN, MRN complex, radiotherapy, biomarker, aCGH

## Abstract

Despite the use of clinical prognostic factors (PSA, T-category and Gleason score), 20-60% of localized prostate cancers (PCa) fail primary local treatment. Herein, we determined the prognostic importance of main sensors of the DNA damage response (DDR): MRE11A, RAD50, NBN, ATM, ATR and PRKDC. We studied copy number alterations in DDR genes in localized PCa treated with image-guided radiotherapy (IGRT; n=139) versus radical prostatectomy (RadP; n=154). In both cohorts, NBN gains were the most frequent genomic alteration (14.4 and 11% of cases, respectively), and were associated with overall tumour genomic instability (p<0.0001). NBN gains were the only significant predictor of 5yrs biochemical relapse-free rate (bRFR) following IGRT (46% versus 77%; p=0.00067). On multivariate analysis, NBN gain remained a significant independent predictor of bRFR after adjusting for known clinical prognostic variables (HR=3.28, 95% CI 1.56–6.89, Wald p-value=0.0017). No DDR-sensing gene was prognostic in the RadP cohort. In vitro studies correlated NBN gene overexpression with PCa cells radioresistance. In conclusion, NBN gain predicts for decreased bRFR in IGRT, but not in RadP patients. If validated independently, Nibrin gains may be the first PCa predictive biomarker to facilitate local treatment decisions using precision medicine approaches with surgery or radiotherapy.

## INTRODUCTION

Men with localized prostate cancer (PCa) are categorized in risk groups that reflect increasing treatment failure rates and prostate cancer specific mortality (*e.g.* low, intermediate or high) on the basis of pathologic Gleason score (GS), pre-treatment serum prostate specific antigen (PSA, measured in ng/mL) and clinical T-category (TNM staging system) [1-2]. Despite clinical classification, intermediate and high-risk PCa are characterized by significant heterogeneity with respect to clinical outcome after image-guided radiotherapy (IGRT) or radical prostatectomy, with biochemical relapse-free rates (bRFR) ranging from 40 to 80% [3]. This suggests that other pathologic, genomic or molecular factors could further triage patients into responders and non-responders by developing novel prognostic (treatment-independent) or predictive (treatment-dependent) biomarkers.

Malignant progression to the metastatic state is associated with increasing oncogene activation, tumor suppressor gene inactivation and increased genomic instability [4]. These genetic alterations may accumulate due to abnormal DNA damage responses (DDR) in the sensing and repair of DNA damage [5-6]. An abnormality in the DDR pathway could lead to cancer progression and metastases and/or affect the relative sensitivity of a tumour to radiotherapy, in which daily doses of photon radiation lead to DNA double-strand breaks (DSBs), and other DNA damages. Indeed, *in vitro* experiments support the hypothesis that differential prostate cancer cell radiosensitivity and genetic instability are correlated to differential DDR integrity [7-8].

The earliest and fundamental step in the process of the DDR is the recruitment of sensing and repair machinery to the sites of DNA damage [9]. The initial protein effectors of the DDR are the MRN complex (MRE11, RAD50 and NBS-1/Nibrin encoded by the *MRE11A, RAD50* and *NBN* genes, respectively) and the DNA damage dependent, PI3-like kinases: ATM, ATR and DNA-PKcs (encoded by the *ATM, ATR* and *PRKDC* genes, respectively). *ATM* and *NBN* mutations have also been suggested to confer increased risk of prostate cancer [10-11] and disease aggression [12-13]. To date, a systematic analysis of copy number alterations (CNAs) within the initial DDR sensor genes (*MRE11A*, *RAD50*, *NBN*, *ATM*, *ATR*, *PRKDC*) as potential prognostic biomarkers has not been explored.

Herein, we collated the alterations in DDR sensors within localized prostate cancer, and show that copy number gains in the *NBN* locus is a novel adverse and independent factor for outcome following radiotherapy, but not following radical prostatectomy, in localized PCa. We discuss the use of *NBN* status to intensify therapy in the context of personalized PCa medicine.

## RESULTS

### CNAs of DDR genes are common in localized PCa

**Figure 1:**
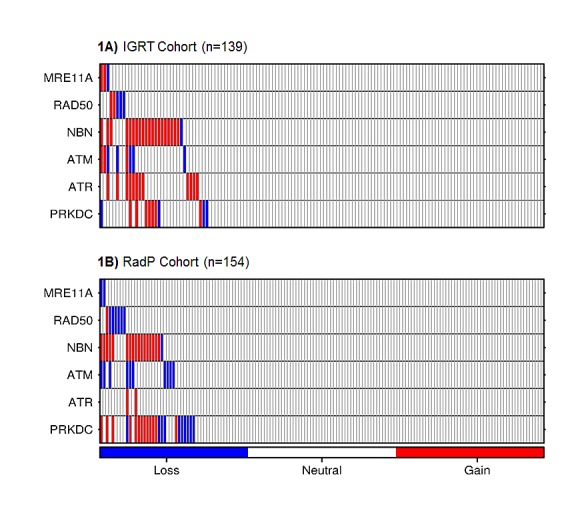
Heatmap of CNAs in DDR genes in tumour biopsies from the IGRT (1A) or RadP (1B) cohorts Each bar represents a single patient. Gains and losses are represented by red and blue bars, respectively.

We initially determined whether DDR-sensing genes (*MRE11A*, *RAD50*, *NBN*, *ATM*, *ATR* and *PRKDC*) exhibited allelic gains or losses in our cohort. Upon evaluation of 139 pre-IGRT biopsies by aCGH, allelic changes were observed predominantly in *NBN* (15.1%), *ATR* (8.6%), *PRKDC* (7.9%) and *ATM* (5.8%). The surgical cohort showed a similar proportion of allelic changes in the *NBN* (11.6%) and *ATM* (6.5%) loci but higher in *PRKDC* (14.9%) and lower in *ATR* (1.3%) loci. *RAD50* and *MRE11A* showed less than 5% of aberrations within each cohort (see Figures 1A/B). CNA distributions in these six DDR genes were not statistically different across any of the clinical prognostic variables of pretreatment PSA, T-category and GS (data not shown).

To determine how hits within the DDR sensor genes relate to each other, we explored the co-segregation and mutual exclusivity patterns of allelic changes in these six loci. To increase statistical power, we combined both treatment cohorts (*pooled cohort*) for this analysis. Five gene-pairs were co-altered in more patients than expected by chance (Fisher's exact test, p < 0.001, Bonferroni correction): *ATM-ATR, ATM-MRE11A, NBN-ATM, NBN-ATR* and *NBN-PRKDC* (Supplemental Figure 1). No aberrations in DDR-sensing genes were mutually exclusive. We conclude that *ATM* and *NBN* alterations can associate with the other DDR alterations in PCa.

### Patients with *NBN* CNAs show increased genomic instability

Considering that DDR genes play a role in genomic integrity, we used the percent genome aberration (PGA) to describe the instability within a patient's tumour genome.

**Figure 2:**
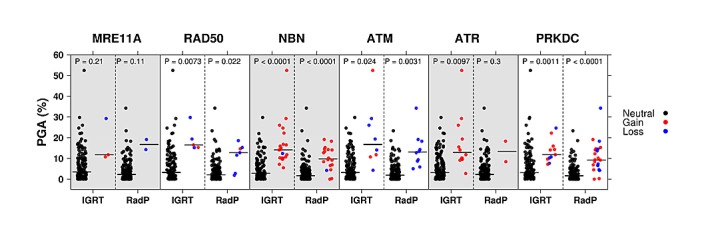
PGA as a function of DDR genes copy number status for IGRT and RadP cohorts Each dot represents an individual patient with black, red and blue dots representing copy number neutral, loss, or gain respectively. The horizontal black line denotes the median PGA for each group. In both the IGRT and RadP cohorts, DDR genes CNAs associate with increased genomic instability (PGA) compared to patients with copy number neutral DDR gene status (p-values are shown; Mann-Whitney-U-test).

*NBN* exhibited the strongest association of a DNA damage response-sensing gene with PGA in both RadP and IGRT cohorts (see Figure 2). Using the *pooled cohort*, CNAs in all six DDR-sensing genes remained significantly correlated with higher PGA, independent of the clinical prognostic variables (pretreatment PSA, T-category and GS) (Supplemental Table 1).

### Patients with CNAs in DDR genes are more likely to relapse after IGRT

**Figure 3:**
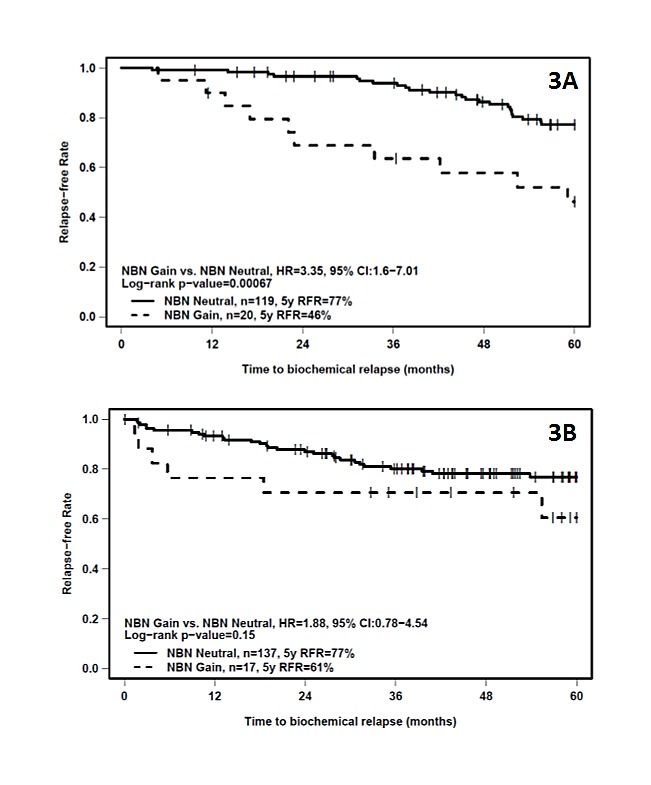
Kaplan-Meier plots of bRFR versus time to recurrence showing the effect of differential *NBA* CNA status (neutral *vs*. gain) in the IGRT (3A) or RadP (3B) treatment cohorts

We next examined the impact of allelic changes in the DDR loci on clinical outcome based on biochemical recurrence (BCR). The clinical characteristics the IGRT cohort are shown in Table 1. At five years, 34 patients (24.5%) experienced biochemical failure as per the Phoenix definition. In this predominantly intermediate-risk group cohort, only PSA remained as a significant clinical prognostic factor for BCR based on univariate analysis (p-value = 0.0035) (see Supplemental Figure 2A). We tested DDR genes CNAs (gains or losses) for prognostic capability. Only *NBN* was prognostic for bRFR at 5 years on univariate analysis (bRFR 46% vs. 77%, log-rank p-value = 0.00067; HR= 3.35; 95% CI: 1.6 – 7.01; see Figure 3A). On multivariate analysis, *NBN* gains remained a significant independent predictor of bRFR after adjusting for PSA, T-category and GS (HR = 3.28, 95% CI 1.56 – 6.89, Wald p-value = 0.0017; see Table 2). *NBN* gains remained a significant predictor of bRFR even within low-risk patients (log rank p-value = 0.0037; Supplemental Figure 3); although the number of low-risk patients with NBN gains is low (4 patients).

**Table 1:** Clinical characteristics of IGRT and RadP cohorts

	IGRT cohort	RadP cohort	How was used in the analysis
	N=139 (%)	N=154 (%)	
**T-category** T1 T2 T3	50 (36%) 89 (64%) -	79 (51.3%) 66 (42.9%) 9 (5.8%)	T1 vs T2-T3
**Gleason-score** 5 6 7 8 9	- 34 (24.4%) 98 (70.5%) 7 (5%) -	2 (1.3%) 82 (53.3%) 53 (34.4%) 11 (7.1%) 6 (3.9%)	GS 5-6 vs 7-9
**Pretreatment-PSA (ng/mL)** Median Range <10 10-20 >20	8.0 0.9 – 33 92 (66.2%) 41 (29.5%) 6 (4.3%)	6.265 1.15 – 506 115 (74.7%) 24 (15.6%) 15 (9.7%)	PSA <10 vs > 10
**NCCN Risk Group** (Clinical staging) Low Intermediate High	19 (13.6%) 107 (77.0%) 13 (9.4%)	45 (29.2%) 79 (51.3%) 30 (19.5%)	-
**Hormone therapy** Neo-adjuvant	35 (25.2%)	7 (4.5%)	-
**RT dose (Gy/fraction)** 60/20 66/22 70/35 75.6/42 78/39 79.8/42 Mean equivalent dose= 75.9Gy	15 (10.8%) 3 (2.1%) 1 (0.7%) 38 (27.3%) 3 (2.1%) 79 (56.8%)	NA	-
**BCR (5yrs)** Yes No	34 (24.5%) 105 (75.5%)	34 (22.1%) 120 (77.9%)	-

**Table 2:** Multivariable analysis in the IGRT cohort of the additional effect of *NBN* gains on bRFR when adjusted for clinical variables (PSA, Gleason score and cT-category)

Variable	Hazard Ratio	95% CI		Wald p-value
**NBN**				
Gain	3.28	1.56	6.89	0.0017
Neutral	1.00	--	--	--
**PSA**				
<10	1.00	--	--	--
≥10	2.93	1.48	5.81	0.0020
**T-category**				
T1	1.00	--	--	--
T2	0.91	0.44	1.86	0.79
**Gleason Score**				
6	1.00	--	--	--
≥7	0.89	0.41	1.93	0.77

We next interrogated the surgically treated RadP cohort (n=154). At five years after surgery, thirty-four patients (22.1%) experienced biochemical failure. All three known clinical prognostic variables remained significant predictors of biochemical outcome (Supplemental Figure 2B). On univariate analysis, *NBN* gain did not have significant prognostic value (HR = 1.88, 95% CI 0.78 – 4.54, log-rank p-value = 0.15) (see Figure 3B). To further evaluate the potential prognostic role of NBN in this cohort, we explored the impact of mRNA abundance and its known partner *KPNA2* on treatment outcome. In the 108 RadP patients with matched CNA and mRNA information, we found a significant association between *NBN* copy-number gain and NBN mRNA abundance (Student's t-test, p-value = 0.0074; Supplemental Figure 4A). However, neither *NBN* nor *KPNA2* mRNA abundance (stratified by median expression) correlated with bRFR (Supplemental Figure 4B/C).

### *NBN* status and expression associates with radioresistance *in vitro*

**Figure 4:**
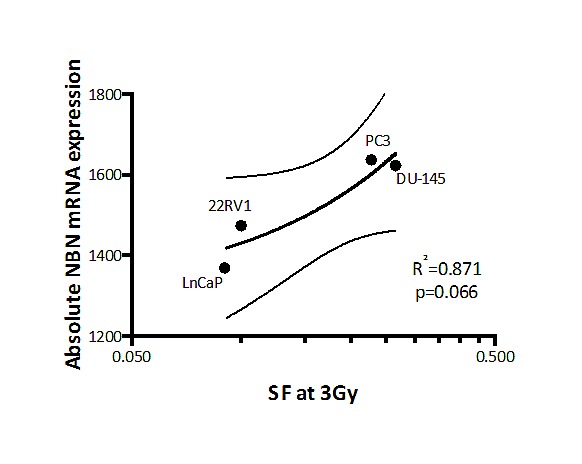
Normalized *NBN* mRNA abundance plotted against relative radioresistance (clonal surviving fraction after 3 Gy; SF3Gy) Linear regression (continuous line) and 95% confidence interval (doted lines) are shown. A correlation is observed (R-square = 0.871) with a significant trend in the p-value of the slope (p=0.066). For PrEC cell line, only clonogenic survival fraction after 2Gy (SF2Gy) was available, therefore it was not included in the linear regression calculation.

Given the potential additional role of *NBN* gains as a predictive factor for outcome after IGRT, we determined the correlation between absolute DDR gene mRNA expression and intrinsic clonogen radioresistance in a panel of prostate cell lines (see Supplemental Figure 5). Of the six DDR genes, only *NBN* gene expression showed a positive correlation with increasing clonogen radioresistance (*e.g.* increasing SF_3Gy_; r^2^ = 0.871, p-value of slope = 0.066; see Figure 4). This data suggest a role for NBN gains in intrinsic tumour resistance to radiotherapy, consistent with its poor prognostic impact in IGRT-treated patients.

## DISCUSSION

To our knowledge, this is the first study to describe the frequency and importance of alterations in genes that are initial sensors within the DDR pathways within PCa patients. We particularly were interested in the potential effect of abnormalities in genes involved in the initial DNA damage response-sensing for IGRT patients who receive daily doses of DNA DSBs as part of their treatment. Our main finding, that an altered DDR is predictive for IGRT outcome, is important given our results are based on a genomic test using pre-treatment biopsies which are used to direct primary management decisions of localized PCa. This prognostic effect was not observed in surgery patients in whom DDR responses are probably irrelevant.

If our IGRT-based observation with *NBN* gains is validated in other independent and/or large prospective cohorts using aCGH or FISH (fluorescence in situ hybridization, using a NBN-specific probe), this could become the first predictive test for selecting patients to undergo surgery (rather than IGRT alone) or undergo IGRT treatment intensification (*e.g.* further radiotherapy dose-escalation or the additional use of systemic agents).

We confirmed our hypothesis that aberrations in DDR genes would associate with unstable PCa genomes. At present, it is not known whether *NBN* gains, are drivers or passengers within PCa progression. It should be noted that the *NBN* locus in on chromosome 8q which is one of the most altered areas of the genome in our patient cohort, and therefore *NBN* gains may strongly relate to other gene loci abnormalities (*e.g.* c-Myc). We cannot yet conclude that *NBN*'s effect is gene-specific (e.g. prognostic at the mRNA and/or protein level) or rather a reflection of an important role as a surrogate of genomic instability; results from ongoing multimodal genomic studies (ICGC and TCGA consortia) may help clarify *NBN* gains linkage to specific CNAs, mutations or gene rearrangements. Furthermore, a recent publication form the TCGA consortia in colorectal cancer has shown that many CNA-driven increases in mRNA levels do not translate into greater abundance of the corresponding protein [14]. Future research is required to characterize NBN gains functional effect in the DDR of PCa tumours.

Based on previous work suggesting that germline *NBN* mutations predispose to more aggressive phenotypes of disease [13], we speculate that failures in IGRT-treated patients could be related to radioresistance and increased *NBN*-associated genomic instability that leads to greater capacity for micrometastatic spread. Neither *NBN* gain nor *NBN* mRNA expression (or expression of its partner, *KPNA2*) was prognostic in the surgery cohort; consistent with previous publications on Nibrin expression in surgically treated prostate cancer patients [15-16]. The only current predictive DDR biomarker for radiotherapy response is MRE11 protein expression in bladder cancer [17], which has been independently validated [18]. However, *MRE11A* alterations were not frequent (nor prognostic) in our PCa cohorts.

We acknowledge a number of limitations to the current study. First, our biomarker and outcome analyses are based on a single TRUS-guided biopsy to the dominant lesion; therefore the known intraprostatic heterogeneity and differential biology of multifocal disease might be underestimated [19]. However, the biopsies were taken from the dominant (index) lesion in each patient, which usually correlates with the majority of local treatment failures [20]. Also, our radiotherapy and surgical cohorts differ slightly in the distribution of clinical prognostic features and duration of follow-up, which could reduce the power to detect significant results in the DNA-based prognosis analyses for the surgical cohort. However, as stated above, protein expression for NBN is not prognostic in other surgical series [15-16].

In conclusion, our study suggests that gain of *NBN* is a potentially novel predictive factor of IGRT failure, and patients should consider surgery if their tumour harbours this CNA. If validated, this would be the first outcome-associated predictive biomarker for local treatment in localized PCa. IGRT patients whose tumours harbour *NBN* gains should be assessed for additional systemic therapies to improve outcome [21].

## MATERIALS AND METHODS

### Study Patient Cohorts

DNA samples for array comparative genomic hybridization (aCGH) analysis were derived from pre-treatment frozen biopsies in patients undergoing radical image-guided radiotherapy (REB#00-0443-C; the Princess Margaret Cancer Centre), as previously described [22]. The clinical target volume (CTV) encompassed the prostate gland alone. The planning target volume (PTV) was defined by a 10 mm expansion around the CTV except posteriorly in which the margin was 7 mm. All patients were treated with 6-field conformal or intensity modulated image-guided radiotherapy. The radiotherapy dose was escalated over the period of accrual in a series of separate Phase I-II studies (see Table 1). Some patients received short-term, neoadjuvant and concurrent anti-androgen treatment (bicalutamide 150 mg po od). None of the patients received LHRH agonists/antagonists nor adjuvant androgen deprivation therapy. Staging bone scans and computed tomography (CT) were not routinely carried out on low- and intermediate-risk disease patients. Patients were followed at 4-6 monthly intervals after completing treatment with clinical examination and PSA measurement. Additional tests and the management of patients with recurrent disease were at the discretion of the treating physician.

Sufficient tumour for aCGH analysis was identified in 139 patients who also had long term follow-up information pertaining to biochemical outcome (see Table 1). The median follow-up of surviving patients was 7.9 years following the start of treatment, with 62 (44.6%) patients experiencing biochemical failure during follow-up.

We interrogated the publicly-available Memorial Sloan Kettering Cancer Center Prostate Cancer database [23] for aCGH data pertaining to 154 PCa tumours of patients treated by radical prostatectomy (RadP). Gene-level CNA calls were collapsed to losses or gains. Patients were placed into low-, intermediate-, and high-risk NCCN categories [2] based on pre-surgery clinical staging. (see Table 1). The median follow-up for this cohort was 4.8 years, with 38 patients (24.7%) experiencing biochemical failure during the entire follow-up. For selected RadP cohort patients (108 out of 154), we also used this database to compare mRNA abundance to allelic gain or loss in DDR genes.

### Array Comparative Genomic Hybridization (aCGH) for CNA Calls

For the IGRT cohort, DNA extraction, labeling and hybridization onto arrays containing 26,819 bacterial artificial chromosome (BAC)-derived amplified fragment pools was carried out as described previously [24]. The resulting data set was normalized using a stepwise normalization procedure [25]. This data is available in NCBI's Gene Expression Omnibus with accession number GSE41120. Areas of aberrant copy number were identified using a robust Hidden Markov Model [26] and classified as either loss, neutral or gain for all clones. The liftOver tool from UCSC was used to map the copy number segments to the hg19 human genome build. Fragments overlapping centromeres, telomeres, or other gaps in the hg18 build were trimmed conservatively (regions were shortened rather than elongated). To generate contiguous CNA regions, probe-based CNA calls were collapsed with neighbouring probes within the same chromosome with the same copy number. CNA regions with only one supporting probe were removed. In addition, any CNAs found entirely within centromeres or telomeres, as defined by the UCSC ‘gap’ table, were removed. CNA regions were intersected with a merged and collapsed version of the RefSeq gene annotation (GRCh37/hg19) to generate gene-based CNA calls. This gene list was further filtered to match the published gene list from the radical prostatectomy (RadP) cohort (n = 17,603).

For both IGRT and RadP cohorts, percent genome aberration (PGA) was used as a measure of genomic instability and defined as the cumulative size of the genetic alterations found in each patient DNA sample divided by the total size of the human genome as previously described [27]. To account for the fact that a gene may be found in a large aberration (which can skew PGA values), we removed chromosomal effects from PGA-association calculations for each gene. For example, for *MRE11A* (which is on chromosome 11), we calculated PGA using chromosomes 1-10 and 12-22 only. Thus, if *MRE11A* is truly associated with PGA, the effect should be observed throughout the genome and unaffected by this adjustment. The raw PGA and the adjusted-PGA are highly correlated (Supplemental Figure 6) and as such, we show adjusted-PGA values throughout the manuscript.

### mRNA Expression and *In Vitro* Radiosensitivity Assays

To corroborate the findings at the CNA level in the surgery cohort, mRNA abundance data for the RadP cohort was also considered for candidate genes. We focused on the 108 patients with primary disease that were previously studied at the mRNA and CNA level [23]. Raw CEL files were downloaded from GEO (accession GSE21034) and normalized with RMA [28], as previously described [29].

Clonogenic assay data on radiation surviving fraction at 3Gy (SF_3Gy_) for PCa cells (PrEC, LNCaP, 22RV1, PC-3, and DU-145) were determined as previously published[22]. All cell lines were obtained from American Type Culture Collection and authenticated by short tandem repeat DNA profiling. Nuclear and cytoplasmic RNA was extracted and assayed with the NanoString nCounter platform. Gene-specific mRNA abundance was determined for *MRE11A*, *RAD50*, *NBN*, *ATM*, *ATR* and *PRKDC* genes, housekeeping genes (β-microglobulin, β-actin, and GAPDH). As recommended, positive control probes (6), geometric mean of housekeeping gene expression and negative control probes (8) were used to normalize the data.

### Statistical Analyses

Patients with copy number gain and/or loss in each of the six DDR genes (*MRE11A*, *RAD50*, *NBN*, *ATM*, *ATR* and *PRKDC*) were compared to patients with copy neutral status for distribution of clinical variables of interest and treatment outcome. Chi-squared tests were used to compare the proportion of patients between CNA groups for clinical variables (PSA, T-category and GS). Association of CNA status of DDR-sensing genes with PGA was assessed by Student's t-tests, and a multiple linear regression model was used to evaluate the effect of DDR genes on PGA adjusting for clinical variables (PSA, T-category and GS). Fisher's exact test was used to assess mutual exclusivity and co-segregation of pairs of DDR-sensing genes. Student's t-tests were calculated to determine the association between copy-number and mRNA abundance. Linear regression was used to describe the relationship between DDR genes mRNA abundance and clonogen radioresistance *in vitro*.

The primary clinical outcome variable for both cohorts was time to biochemical recurrence, censored at five years. Cox proportional hazards regression models were used for univariate and multivariate models to assess the prognostic ability of DDR genes on biochemical relapse-free rate adjusting for known clinical prognostic factors. (PSA <10 vs. >=10; T1 vs. T2-T3; Gleason score 5-6 vs. 7 or higher). The proportional hazards assumption was checked by investigating the Schoenfeld residuals. Five-year relapse-free rates were estimated using the Kaplan-Meier method. The additional utility of any copy number alteration relative to clinical parameters was described by the use of c-statistic, net reclassification index (NRI), and integrated discrimination improvement (IDI) [30]. The c-statistic presents the probability that a classification model gives a higher score to a patient that experiences a biochemical relapse by 5 years compared to a patient censored at five years. Thus, in the clinical setting, adding one biomarker to a validated clinical model may not change the classification of an event case to a non-event case. NRI represents another informative measure of added value, which considers whether adding NBN to the basic clinical model increases the model's risk scores for those patients that experienced a biochemical recurrence or decreases the risk scores for patients that were censored at 5 years, respectively. In turn, IDI calculates the improvement in sensitivity without changing specificity by calculating the difference in survival probability for each patient from the clinical model to the model with *NBN* gain incorporated.

All tests are two-sided and statistical significance was assessed using p<0.05. Multiple testing correction was applied where appropriate with the Bonferroni method, as specified in the text. All statistical analyses were performed using the open-source software R (v3.0-1). The survival package (v2.37-4) was used to plot Kaplan-Meier curves and run Cox proportional-hazards regression models; the lattice (v0.20-15) and latticeExtra (v0.6-24) packages were used for data visualization.

## SUPPLEMENTARY MATERIAL, FIGURES AND TABLES


