# Supplementary material for: NBN gain is predictive for adverse outcome following image-guided radiotherapy for localized prostate cancer
Source: Oncotarget. 2014 Aug 27;5(22):11081–90. doi: 10.18632/oncotarget.2404 (PMC4294365; doi:10.18632/oncotarget.2404)
Supplement: Supplementary file 1 [file oncotarget-05-11081-s001.pdf]

NBN gain is predictive for adverse outcome following image-guided radiotherapy for localized prostate cancer

Supplementary Material

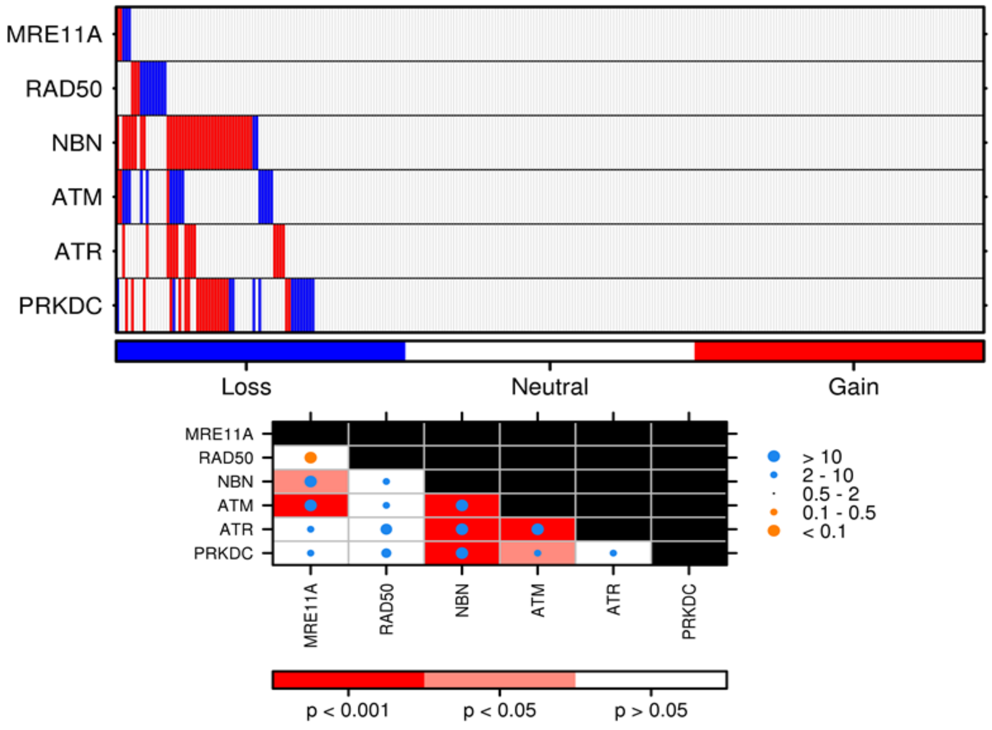

Supplemental Figure 1 : Distribution of CNAs in DDR genes in tumour biopsies for the pooled (IGRT plus RadP) cohort. Each bar represents a single patient. Gains and losses are represented by red and blue bars, respectively. Table shows mutual exclusivity/co-occurrence analysis for pairs of DDR genes. Fisher's exact test p-values are represented by square's background color. Odds ratios ranges are symbolized by coloured circles proportional to the strength of the tendency of mutual exclusivity (0.1-0.5 and <0.1) or co-occurrence (2-10 and >10). After multiple testing correction (Bonferroni), CNAs in gene pairs *ATM-ATR, ATM-MRE11A, NBN-ATM, NBN-ATR and NBN-PRKDC* exhibited a higher tendency towards co-occurrence than expected by chance (Fisher's exact test, p<0.001).

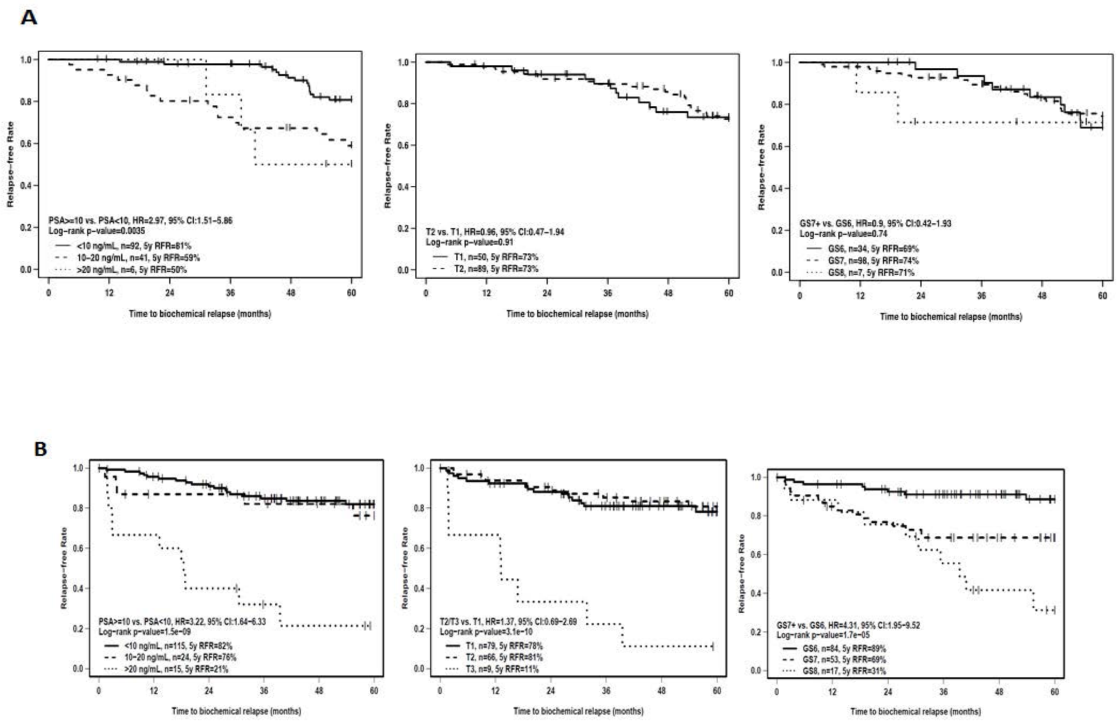

Supplemental Figure 2: Univariate Kaplan-Meier plots of bRFR based on differential PSA (<10 vs >=10), clinical T-category (T1 vs T2-T3) and Gleason score (5-6 vs 7-9) in the IGRT (A) and RadP cohorts (B).

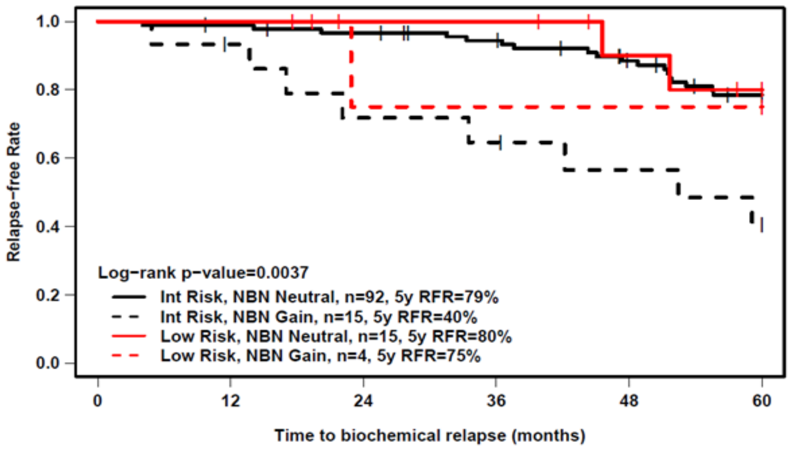

Supplemental Figure 3: Univariate Kaplan-Meier plots of bRFR in low-and intermediate-risk group categories based on differential *NBN* status in the IGRT cohort.

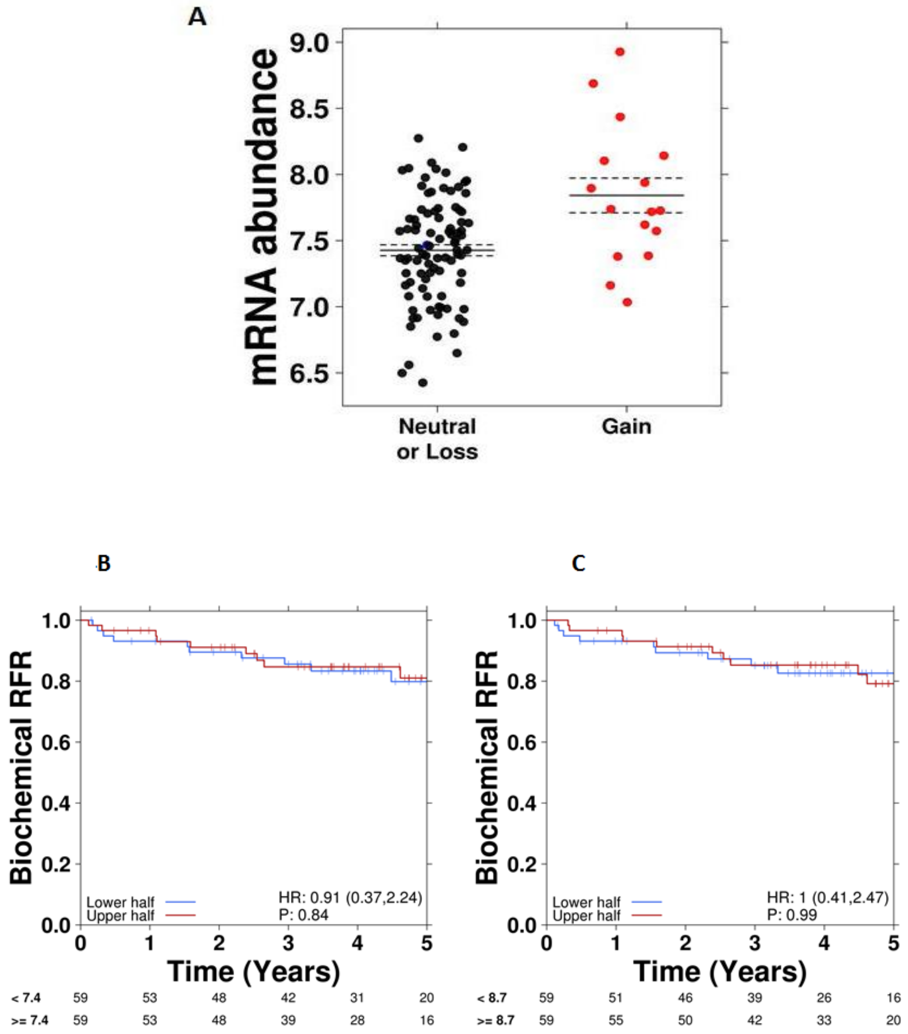

Supplemental Figure 4: (A) mRNA expression of *NBN* in RadP patients plotted according to *NBN* copy number status. The solid black bars and dashed black lines denote the group mean and standard error of the mean, respectively. Patients with gains (n = 16) have significantly higher mRNA abundance than the remaining patients (n = 92, including one patient with a deletion shown in blue) (p=0.0074; Student's t-test). Univariate Kaplan-Meier plots of bRFR based on differential expression levels (mRNA abundance stratified by median) of *NBN* (B), and *KPNA2* (C). Analyses were performed on a subset of RadP cohort patients with mRNA microarray information available (n=108).

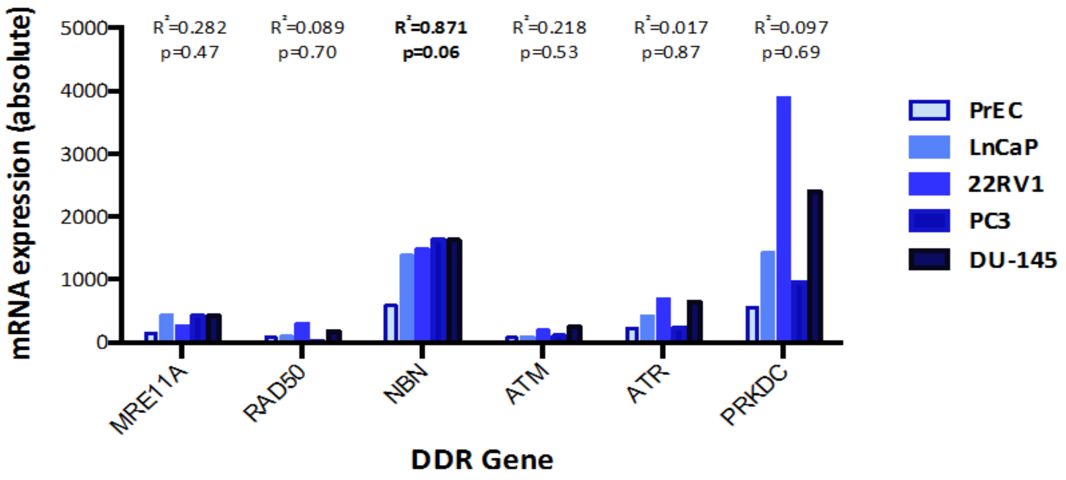

Supplemental Figure 5: Normalized mRNA abundance for each of the six DDR genes in the five prostate cancer cell lines. These are arranged by relative radioresistance (lower to higher from left to right, respectively). Linear regression coefficient (R^2) and p-value of the slope for each DDR gene absolute mRNA expression vs. SF3Gy in four cell lines (PrEC excluded) are glossed above the corresponding columns.

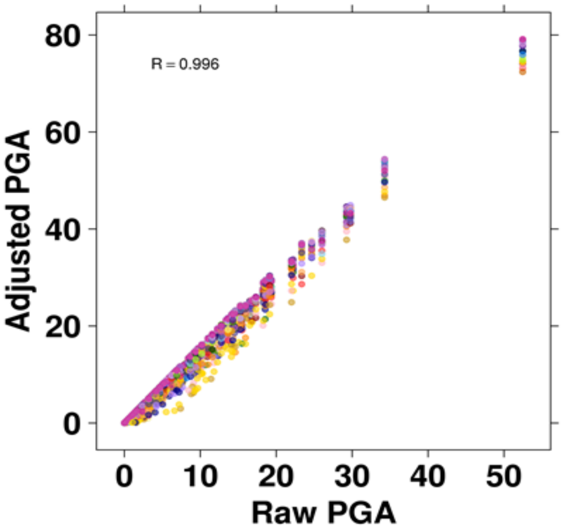

Supplemental Figure 6: Strong correlation between raw and adjusted PGA values (R=0.996; see Methods for details). Each autosomal chromosome is depicted by a different color.

Supplemental Table 1: Percentage genome aberration (PGA) for sub-groups categorized by each DDR gene status and clinical variables (PSA, clinical T-category and GS). Mean PGA is shown for each sub-group. Multiple linear regression model to evaluate the effect of each DDR gene CNAs effect on PGA, adjusted by clinical variables (PSA, T-category and GS): p-values are shown.

		PSA			T-category			Gleason Score			Adjusted association of DDR-gene CNAs and PGA (p-value)
		< 10	10 - 20	≥ 20	T1	T2	T3	≤ 6	7	≥ 8	
**MRE11A**	Neutral	6.47	10.49	12.34	7.28	7.72	11.28	6.56	7.93	11.01	0.00072
	CNA	21.15	25.93	20.71	18.91	42.67	20.71	21.15	17.57	42.67	
**RAD50**	Neutral	6.06	9.7	9.57	6.68	7.23	10.49	5.81	7.54	8.72	8.32×10^{-5}
	CNA	20.89	18.29	21.08	26.13	16.37	13.57	20.02	20.37	23.4	
**NBN**	Neutral	4.7	7.45	11.23	5.69	4.94	10.14	4.33	5.68	7.19	5.7×10^{-11}
	CNA	14.62	17.21	13.37	11.74	19.64	14.69	16.97	13.7	31.14	
**ATM**	Neutral	5.93	9.01	10.36	6.65	6.66	11.28	5.6	7.52	7.93	2.83×10^{-14}
	CNA	21.59	28.3	35.33	19.65	31.43	20.71	24.18	19.14	35.83	
**ATR**	Neutral	5.8	9.67	12.68	7.15	6.38	12.15	5.98	7.25	7.55	2.34×10^{-8}
	CNA	20.77	22.9	6.76	20.24	22.08	NA	36.49	14.25	32.92	
**PRKDC**	Neutral	5.01	8.18	9.29	5.41	6.14	9.41	5.12	6.11	9.19	1.7×10^{-5}
	CNA	10.46	18.46	21.61	13.59	12	20.52	11.25	12.25	15.13	

Supplemental Table 2: Clinical model discrimination with the addition of NBN gains

Model	Statistic	Value	95% CI	p-value
Clinical Model	c-statistic	0.645	0.528-0.761	0.335
Clinical Model + NBN		0.673	0.562-0.785	
Clinical Model	NRI	0.185	-0.074-0.365	0.113
Clinical Model + NBN				
Clinical Model	IDI	0.060	0.0047-0.176	0.026
Clinical Model + NBN				
